# Computational in silico genetic variant prediction tools in cardiovascular disease

**DOI:** 10.1111/anec.13079

**Published:** 2023-08-21

**Authors:** Lydia D. Hellwig, Joaquin Villar, Clesson Turner

**Affiliations:** ^1^ The Henry M. Jackson Foundation for the Advancement of Military Medicine, Inc. Bethesda Maryland USA; ^2^ Center for Military Precision Health Uniformed Services University of the Health Sciences Bethesda Maryland USA; ^3^ Department of Pediatrics Uniformed Services University of the Health Sciences Bethesda Maryland USA; ^4^ Center for Precision Health Research, National Human Genome Research Institute National Institutes of Health Bethesda Maryland USA

Clinical genetic testing for hereditary cardiovascular diseases is recommended by many cardiovascular groups (Musunuru et al., 2020; Wilde et al., 2022). Genetic test results can be important for patient medical management and for the care for family members (Cirino et al., 2017). Appropriate classification of genetic variants is a critical component of this process and ultimately impacts patient and family outcomes (Care et al., 2017; Phillips et al., 2005). The American College of Medical Genetics and Genomics and the Association for Molecular Pathology (ACMG/AMP) created recommendations for the classification of pathogenicity of variants in genes associated with monogenic disease (Richards et al., 2015). These recommendations include defining the criteria for evidence used in classification as well as providing a framework for weighing and combining different types of evidence for the classification. Despite this standardized approach to interpretation, analysis and appropriate classification of variants remain challenging across disease contexts in clinical genetics (McInnes et al., 2021).

In this issue of *Annals of Noninvasive Electrocardiology*, Younis et al. (2023) report that computational genetic variant prediction tools could identify the majority of pathogenic variants in congenital long QT syndrome (LQTS) 1–3. The authors also found that the computational scores did not predict clinical outcomes.

While it is encouraging that the variant prediction tools correlated with pathogenicity in this study, it is also important to note that determination of variant pathogenicity includes multiple types of evidence, including variant prediction evidence. Importantly, computational in silico predictors alone should not be used to classify the pathogenicity of a variant, but can be used as one piece of evidence in the classification of a genetic variant. The ACMG/AMP recommendations specify that using computational predictors are “supporting” level of evidence for or against pathogenicity using criteria PP3 and BP4 (Care et al., 2017). Supporting‐level evidence must be combined with other more substantial lines of evidence to classify the variant. Furthermore, a recent manuscript by Pejaver et al. (2022) provided evidence for redefining how computational tools can be used to provide evidence for or against pathogenicity of variants using the Bayesian adaptation of the ACMG/AMP framework. This work showed that the tools can provide stronger than supporting evidence and the computational tools varied in their ability to reach these levels of evidence. These authors also pointed out that it is important to select a single tool to use for PP3/BP4 missense evidence to avoid biases in results selection.

In addition, although the terms continue to be used interchangeably in the literature, recently, the clinical genetics and genomics community has begun to distinguish the differences between the terms *variant classification* and *variant interpretation*. Variant classification is defined as the process of evaluating pathogenicity of a variant, while variant interpretation refers to the process of clinical integration of the genetic test results with patient clinical characteristics and family history to arrive at a diagnosis (Biesecker et al., 2018). These nuances are challenging in the field and it will continue to be important to carefully define and use such terms clearly in clinical genomic work in the future.

Younis et al. (2023) also found that the computational in silico tools did not predict clinical outcomes and conclude that variant location/functional analysis are needed for more accurate risk interpretation. The potential use of computational in silico tools outside of the variant classification process, such as using these tools as indicators of clinical severity, is much less clear. Accurate assessment of risk interpretation in LQTS and other heritable cardiovascular diseases continues to be challenging, complicated by variable expressivity and incomplete penetrance (Lankaputhra & Voskoboinik, 2021). In addition to additional location/functional data that may help clarify risk stratification among individuals with pathogenic and likely pathogenic variants in associated genes, further population‐based and genotype‐first approaches may also be used to further assess these complex issues related to variant interpretation (Wilczewski et al., 2023).

## AUTHOR CONTRIBUTIONS

All authors (LDH, JV, and CT) were involved in the conception/intepretation, drafting or revising, and provided final approval of the manuscript submitted.

## CONFLICT OF INTEREST STATEMENT

The authors have no conflict of interest to disclose.

## DISCLAIMER

The opinions and assertions expressed herein are those of the author(s) and do not reflect the official policy or position of the Uniformed Services University of the Health Sciences or the Department of Defense. The opinions and assertions herein are those of the author(s) and do not reflect the official policy or position of the Henry M. Jackson Foundation for the Advancement of Military Medicine, Inc. or the National Institutes of Health.

## Data Availability

Data sharing is not applicable to this article as no new data were created or analyzed in this study.
